# Effects of reduced gluten intake through the inclusion of yellow pea-based products on abdominal symptoms in habitual wheat consumers: a non-randomized clinical trial

**DOI:** 10.1007/s00394-026-04097-2

**Published:** 2026-08-29

**Authors:** Hitoshi Shimbo, Mamoru Ito, Yuto Aoki, Joto Yoshimoto, Mikiya Kishi, Jiro Saito, Yukihiro Ohya, Masashi Nakamura, Senju Hashimoto, Akiko Yagami

**Affiliations:** 1 Central Research Institute, Mizkan Holdings Co., Ltd., Handa-shi, Japan; 2 Medical Station Clinic, Meguro-ku, Japan; 3 Department of Allergology, Fujita Health University School of Medicine, Nagoya, Aichi Japan; 4 Fujita Health University General Allergy Center, Nagoya, Aichi Japan; 5 Department of Gastroenterology, Fujita Health University Bantane Hospital, Nagoya, Aichi Japan

**Keywords:** Non-coeliac gluten sensitivity, Gluten-reduced diet, Yellow pea, Abdominal symptoms, Gut microbiota, Quality of life

## Abstract

**Purpose:**

Increasing attention has been paid to individuals who report gastrointestinal symptoms following wheat consumption. In addition to wheat allergy and celiac disease, non-celiac gluten sensitivity (NCGS) has been proposed as a clinical entity. Despite persistent symptoms, many affected individuals continue to consume wheat-containing foods. We aimed to investigate the impact of replacing wheat-based foods with gluten-reduced foods, specifically yellow pea pasta (YPP), bread (YPB), and chips (YPC), on the abdominal symptoms, quality of life (QOL), and gut microbiota in healthy individuals who habitually consume wheat frequently.

**Methods:**

Thirty men and women aged 18–64 years with habitual high wheat intake were enrolled and divided into two groups based on the presence or absence of abdominal symptoms. Participants followed a 4-week gluten-reduced diet intervention using YPP, YPB, and YPC. Abdominal symptoms, QOL, gut microbiota, and selected blood biomarkers were evaluated before and after the intervention.

**Results:**

The intervention led to significant improvement in abdominal distension, abdominal pain or discomfort, feeling of incomplete evacuation**,** hard stools, and urgent need for defecation. Baseline QOL differences between groups were no longer evident. Analysis of gut microbiota revealed maintenance and/or an increase in short-chain fatty acid-producing bacteria, with significant correlations observed between specific bacterial shifts and symptom improvement.

**Conclusions:**

Yellow pea–based products (YPP, YPB, and YPC) may represent a promising dietary strategy for alleviating abdominal symptoms in individuals with high wheat consumption. This study was registered in the UMIN Clinical Trials Registry (UMIN Trial ID: UMIN000056466).

**Supplementary Information:**

The online version contains supplementary material available at https://doi.org/10.1007/s00394-026-04097-2.

## Introduction

Since the postwar period, wheat consumption in Japan has increased markedly due to factors such as convenience and palatability. Specifically, annual per capita flour consumption rose from approximately 10 kg immediately after World War II to about 32 kg in fiscal year 1967 and has continued a gradual upward trend over subsequent decades [1]. These findings indicate substantial changes in Japanese dietary habits.

In recent years, evidence has indicated that a subset of individuals consuming wheat-based products, such as bread and noodles, experience gastrointestinal symptoms, including abdominal distension, abdominal pain, diarrhoea, and constipation following ingestion, despite the absence of coeliac disease or wheat allergy. This condition is referred to as non-coeliac gluten sensitivity (NCGS). It has gained clinical attention because it does not only cause physical discomfort but also contributes to psychological stress and reduced quality of life (QOL) [2, 3]. Notably, symptom improvement has been observed in some cases following the introduction of a gluten-free diet (GFD) [4]. However, the etiology of NCGS remains poorly understood. One proposed mechanism is that gluten increases intestinal permeability, thereby contributing to the development of symptoms in individuals with NCGS [5]. Furthermore, recent evidence suggests that FODMAP, particularly fructans contained in wheat, may also play a role in symptom generation [6] Wheat is a major dietary source of fructans and is therefore classified as a high-FODMAP food [7]. Similarly, the yellow peas used in the present study have been reported to contain substantial amounts of galacto-oligosaccharides (GOS), another class of FODMAP [8].

Currently, no established biomarkers exist for the diagnosis of NCGS. From a clinical perspective, diagnosis should follow a systematic protocol comprising the exclusion of coeliac disease and wheat allergy, documentation of symptom improvement following at least 6 weeks of GFD, and, where feasible, confirmation of reproducibility through double-blind crossover trials [4]. However, these procedures are time-consuming and costly, and double-blind crossover trials are rarely conducted in routine clinical practice. Moreover, NCGS symptoms often overlap with those of irritable bowel syndrome (IBS) and functional dyspepsia (FD) [9], with approximately 20% of individuals with NCGS reported to fulfil the Rome IV diagnostic criteria for IBS [10]. This symptom overlap complicates clinical differentiation, potentially leading to underdiagnosis of NCGS and resulting in some individuals unknowingly having the condition while continuing to consume wheat-containing foods on a regular basis.

GFD is an established therapy for coeliac disease and may also benefit individuals with non-coeliac gluten sensitivity (NCGS) [11]. However, adherence to a gluten-free diet has been reported to reduce dietary fiber intake [12]. Furthermore, several studies have demonstrated that strict gluten exclusion can alter gut microbiota composition, reducing beneficial bacteria, such as butyrate-producing bacteria [13, 14]. This highlights the need for alternative dietary strategies that reduce gluten intake while maintaining sustainability without compromising the intestinal environment. The dissemination of such foods could help alleviate symptoms and enhance QOL among individuals with NCGS or those who unknowingly experience wheat-related abdominal symptoms.

The yellow pea (*Pisum sativum *L*.*), cultivated globally, has recently gained attention as a functional food source, particularly due to its high dietary fibre content, along with other nutritional components such as proteins and minerals, which are beneficial to human health [15, 16]. We developed highly palatable yellow pea-based pasta (YPP), bread (YPB), and chips (YPC), which contain no wheat or gluten. The consumption of YPP once daily for 4 weeks has been reported to increase short-chain fatty acid (SCFA)-producing bacteria, including *Bifidobacterium longum* and *Ruminococcus bromii* [17]. Given their gluten-free and fibre-rich composition, these products may improve gut microbiota profiles and help alleviate abdominal symptoms.

Therefore, we aimed to examine the effects of a dietary intervention that reduces gluten intake using YPP, YPB, and YPC in individuals with habitual high wheat consumption, classified into two groups based on the presence or absence of abdominal symptoms.

## Materials and methods

### Study design

This open-label study was conducted between December 2024 and February 2025 at the Medical Station Clinic in Japan. As the study was exploratory in nature, no statistical power calculation was conducted. The sample size was set at 30 participants. Participants were stratified into two groups based on the presence or absence of abdominal symptoms: a symptomatic group (AS+ group:* n* = 15), who met the diagnostic criteria for IBS or FD, or for both according to Rome IV criteria, and an asymptomatic group (AS− group: n = 15), who met neither criterion.

This study adhered to the ethical principles of the Declaration of Helsinki. The study protocol was approved by the Institutional Review Board of the Medical Station Clinic (approval number: 241212-3). All participants provided written informed consent after receiving detailed explanations of the study procedures, the voluntary nature of participation, and their right to withdraw at any time. This study was registered in the UMIN Clinical Trials Registry (16/12/2024 UMIN trial ID: UMIN000056466). The study adhered to the Transparent Reporting of Evaluations with Nonrandomised Designs (TREND) reporting guidelines for nonrandomised trials [18].

### Study participants

Participants with habitual high wheat consumption were screened and recruited through multiple research companies, including JCVN Support Inc. (Tokyo, Japan), Every Inc. (Tokyo, Japan), Q Life Inc. (Tokyo, Japan), 3H Medisolution Inc. (Tokyo, Japan), Sysmore Inc. (Tokyo, Japan), DRC Inc. (Osaka, Japan), LDM Communications Inc. (Kanagawa, Japan), Incrom Inc. (Osaka, Japan), CTSC Inc. (Tokyo, Japan), and Breakthrough Inc. (Tokyo, Japan). Recruitment occurred from December 16 to December 25, 2024, and all follow-up assessments were completed by February 28, 2025.

An online questionnaire survey identified 56 eligible participants who met the inclusion criteria and provided informed consent. Based on pre-enrolment clinical symptom questionnaires, participants were classified into two groups: the AS+ group (*n* = 23) and the AS− group (*n* = 33). The AS+ group comprised individuals who met the diagnostic criteria for IBS and FD or both according to the Rome IV criteria. To ensure clear differentiation between groups and minimise stratification bias, participants in the AS+ group were prioritised for selection based on the number of diagnostic criteria met. After applying exclusion criteria, 15 participants from each group were included in the final intervention cohort.

Participants were excluded from the analysis if meals containing wheat-based processed foods accounted for ≥20% of the total number of meals recorded in the dietary diaries during the intervention period, based on predefined adherence criteria. This percentage was calculated on a meal basis; wheat-based processed foods were considered present if they appeared in a meal, and the proportion was defined as the number of meals containing such foods divided by the total number of meals. Additionally, participants were excluded at the discretion of the principal investigator if confounding factors such as infectious diseases were identified.

Inclusion criteria were as follows: adults aged 18–64 years at the time of informed consent; body mass index (BMI) of 18.5 to < 25 kg/m^2^; and habitual consumption of wheat-based processed foods as staple foods ≥ 14 times per week.

Exclusion criteria were as follows: a self-reported history of physician-diagnosed gastrointestinal disorders (e.g., IBS, FD, celiac disease, inflammatory bowel disease, or other related organic gastrointestinal diseases); the presence of any food allergies (e.g., wheat allergy; excluding hay fever) or a risk of allergen-induced reactions; abnormal blood or cardiopulmonary function tests that could compromise study participation; chronic conditions requiring continuous medication (including herbal medicine, both topical and oral formulations), active treatment of any disease (excluding dry eye and dental caries treatment), or serious disease history requiring pharmacological treatment; pre-study examination values markedly deviating from reference ranges; irregular work schedules (e.g., shift work or night duty); participation in other clinical studies, clinical trials, or human trials within one month before the study; planned travel or business trips (domestic or international) or scheduled events during the intervention period that would hinder the consumption of gluten-free foods; planned lifestyle changes during the study period (e.g., marriage, smoking initiation); habitual dietary intake per meal substantially exceeding the typical amounts; inability to maintain study diaries during the study period; planned pregnancy or lactation during the study; inability to consume the test food during the study period; and any other reasons deemed by the attending physician to render the participant unsuitable for study inclusion.

During the intervention period, participants were instructed not to initiate or regularly consume any medications (e.g., antispasmodics, laxatives, proton pump inhibitors), dietary supplements, or functional foods that could potentially influence the study outcomes. Any use of such products was to be recorded in a diary.

### Intervention

Participants in both groups were instructed to avoid all wheat-containing foods, excluding seasonings, for 4 weeks. To support dietary adherence, the following test foods were provided for each participant, with consumption permitted ad libitum in their habitual living environment: 32 bundles of YPP, 20 packages of YPB, with each containing three varieties (YPB1, YPB2, and YPB3), 30 packages of YPC, and 30 packs of Sato no Gohan (SR). The test food was initially distributed in person at the Medical Station Clinic a few days before the study initiation, and the remainder was delivered to each participant's home by EP Mediate Co., Ltd. via a courier service. Dietary adherence and wheat intake were monitored using daily food diaries. Those who failed to complete their diary were contacted via email or telephone, as appropriate. The energy content and macronutrient composition (energy, protein, fat, carbohydrates, sugar, dietary fibre, and salt equivalents) of each study food are presented in Table 1.

**Table 1:** Nutritional information of the study food (per bundle/per bag/per pack)

Nutrient Composition	YPP	YPB1	YPB2	YPB3	YPC	SR
Energy	262 kcal	115 kcal	170 kcal	145 kcal	155 kcal	294 kcal
Protein	17.0 g	3.7 g	3.5–7.3 g	3.8–6.5 g	4.4–7.6 g	4.2 g
Fat	1.2–2.3 g	6.0 g	5.5–11.0 g	4.4 g	6.6 g	0 g
Carbohydrate	50.4 g	13.0 g	16.8–26.4 g	24.3 g	21.2 g	67.8 g
Sugars	38.6 g	10.0 g	9.1–20.4 g	17.4 g	10.2–15.7 g	–
Total dietary fibre	9.3–14.2 g	3.0 g	5.0–9.5 g	5.6–8.2 g	6.5–9.7 g	–
Salt equivalent	0.01 g	0.2 g	0.29–0.47 g	0.46 g	0.56 g	0 g

YPP, yellow pea pasta; YPB1–3, yellow pea-based bread products 1–3; YPC, yellow pea chips; SR, Sato no Gohan.

### Outcome

Outcomes included changes in clinical symptoms, QOL, biochemical parameters, and gut microbiota composition assessed before and after the intervention.

### Data measurement

Participants underwent comprehensive assessments, including vital signs and anthropometric measurements, blood and stool tests, and completion of a 14-item clinical symptom questionnaire using an 11-point numeric rating scale based on the Salerno Experts' Criteria, a QOL assessment using the SF-36v2 questionnaire, and maintenance of daily diaries documenting dietary intake and lifestyle habits. Participants completed medical interviews with physicians at weeks 0 and 4.

### Vital signs and body composition measurements

At each clinical visit, blood pressure (systolic and diastolic) and heart rate were measured using a fully automated sphygmomanometer (A&D Company Ltd., Tokyo, Japan). Body composition was measured using a fully automated height and weight scale (A&D Company, Ltd., Tokyo, Japan).

### Blood sample

At the baseline clinic visit, 18 mL of blood was collected from each participant by nurses using syringes and blood collection tubes; this was repeated at week 4 clinic visit. Participants fasted for at least 8 h before blood collection time; only water was permitted. Because zinc levels are influenced by diurnal variation, blood collection in the afternoon was prohibited. The following concentrations were measured: interferon-gamma (IFN-γ), interleukin (IL)− 1β, IL-2, IL-4, IL-6, IL-12, IL-17A, and tumour necrosis factor-alpha (TNF-α) using the S-PLEX Proinflammatory Panel 1 (human) (Meso Scale Discovery); IL-13 using the S-PLEX Singleplex Assays (Meso Scale Discovery); and anti-gliadin IgG antibodies using the Human Anti-Gliadin IgG ELISA kit (EPITOPE DIAGNOSTICS). Serum iron, ferritin, and zinc levels were measured by Nikken Seil Co., Ltd. (Tokyo, Japan). Participants who engaged in exercise at intensities beyond their usual daily activity were excluded from cytokine analysis, as such activity may influence inflammatory cytokine expression. Values below the detection limit were assigned a value of zero for statistical analysis [19].

### Questionnaire

Participants completed web-based questionnaires and daily diaries at the following timepoints:

1. Clinical Symptom Assessment
  Clinical symptoms during the preceding week were assessed at weeks 0, 2, and 4 of intervention using a 14-item questionnaire developed based on the Salerno Experts' Criteria [2]. Each item was rated on an 11-point numerical rating scale (0 = no symptoms, 10 = most severe symptoms). Items covered representative NCGS-related abdominal symptoms (abdominal pain or discomfort, nausea or vomiting, abdominal distension, decreased passage of stools, increased passage of stools, loose stools, hard stools, urgent need for defecation, feeling of incomplete evacuation), as well as extraintestinal symptoms (dermatitis, headache, foggy mind, and joint/muscle pain).
2. QOL Assessment
  Health-related QOL was assessed at weeks 0 and 4 using the Japanese version of the SF-36v2 standard edition [20, 21]. It included eight subscales: physical functioning, role physical, bodily pain, general health, vitality, social functioning, role emotional, and mental health. Each subscale score was standardised to a 0–100-point scale [22]. Based on these eight scores, three summary scores were calculated: Physical Component Summary (PCS), Mental Component Summary (MCS), and Role/Social Component Summary (RCS) [23].
3. Daily Diary
  Participants maintained daily web-based diaries to record consumption of test food and wheat-based products (gluten-containing foods), changes in physical condition or lifestyle habits, and any medication use.

### Faecal sampling, DNA extraction, and sequencing

Faecal samples were collected using Mykinso faecal collection kits containing a guanidine thiocyanate solution (Cykinso, Inc., Tokyo, Japan) and stored at 4 ℃. Subsequently, DNA was extracted from the samples using an automated DNA extraction machine (GENE PREP STAR PI-1200A, Kurabo Industries Ltd., Osaka, Japan) following the manufacturer’s protocol. The V1–V2 region of the 16S rRNA gene was amplified using forward primers (16S_27Fmod: TCG TCG GCA GCG TCA GAT GTG TAT AAG AGA CAG AGR GTT TGA TYM TGG CTC AG) and reverse primers (16S_338R: GTC TCG TGG GCT CGG AGA TGT GTA TAA GAG ACA GTG CTG CCT CCC GTA GGA GT) using the KAPA HiFi Hot Start Ready Mix (Roche). Dual-index adapters were ligated to the 16S rRNA gene amplicons with the Nextera XT Index Kit, followed by sequencing on the Illumina MiSeq platform. Each library was diluted to a final concentration of 5 ng/µL, and equal volumes were pooled to obtain a 4 nM mixed library. The DNA concentration of the mixed libraries was quantified by quantitative polymerase chain reaction (qPCR) using the KAPA SYBR FAST qPCR Master Mix (KK4601, KAPA Biosystems) using primers 1 (AAT GAT ACG GCG ACC ACC) and 2 (CAA GCA GAA GAC GGC ATA CGA). Libraries’ preparation followed the Illumina 16S library preparation protocol (Illumina, San Diego, CA, USA). Libraries were sequenced using the MiSeq Reagent Kit v2 or v3 (500 or 600 cycles) and 250- or 300-bp paired-end reads.

### Taxonomy assignment based on 16S rRNA gene sequences

Paired-end reads of the partial 16S rRNA gene sequences were analysed using QIIME 2 (version 2020.8). The data processing and taxonomic assignment followed the standard QIIME 2 pipeline: (1) paired-end reads were joined, filtered, and denoised using DADA2; and (2) taxonomic classification of each amplicon sequence variant was performed using a naïve Bayes classifier trained on the SILVA database (version 138) corresponding to the V1–V2 region of the 16S rRNA gene to determine the identity and composition of the bacterial genera present.

Sequences resolved up to family level (D_4) but unresolved at genus level (D_5), or sequences resolved up to genus level (D_5) but unresolved at species level (D_6), were designated as <family name>; unclassified or <genus name>; unclassified.

### α-diversity and β-diversity analysis

Illumina MiSeq FASTQ reads were subjected to a BLAST homology search against the NCBI 16S microbial database using a Homology Analysis tool (World Fusion Co., Ltd., Tokyo, Japan). Taxonomy classification was performed using the Metagenome@KIN (World Fusion) software. Sequences with an E-value > 0.001 were designated as No BLAST hit, and those with an E-value of > 10E−30 and an identical % < 98% were designated as unclassified hit. Microbial α-diversity was assessed using the Simpson index and Shannon index after excluding no-blast hits and unclassified hit reads. Statistical analysis was performed using the Kruskal–Wallis test.

β-diversity, representing differences in microbial community composition between visits, was assessed using permutational multivariate analysis of variance (PERMANOVA) with repeated measures (adonis2, vegan R package), based on generalized UniFrac distance matrices calculated using weight = 0.5. Within-subject pre–post comparisons were conducted using paired statistical tests, while between-group comparisons were performed using unpaired statistical tests. Principal Coordinates Analysis (PCoA) was performed for visualization, with 95% confidence ellipses plotted for each visit. Statistical significance was determined using 999 permutations.

### Statistical analysis

Data are presented as means with 95% confidence intervals or medians with interquartile ranges. Normality distribution of data was checked by visual inspections of histograms and by Shapiro-Wilks test. To clarify the effects of yellow pea product consumption on clinical symptoms, a subgroup analysis was conducted among participants with an estimated daily intake of ≥ 80 g of yellow pea-containing products (YPP, YPB, and YPC), based on dietary records. This approach was chosen according to prior evidence indicating that daily intake of 80 g of YPP alone for 4 weeks improves faecal microbiota and reduces oxidative stress [17, 24]. Within-group comparisons (between weeks 0 and 4) were conducted using paired t-tests for normally distributed data, while the Wilcoxon signed-rank test was used for non-normally distributed data. Between-group comparisons were conducted using an independent t-test for normally distributed data, while the Wilcoxon rank-sum test was used for non-normally distributed data. Correlations between the changes in clinical symptoms and faecal microbiota abundance in the AS+ group were examined using Spearman’s rank correlation coefficients. All statistical analyses were performed using R version 4.4.3. The significance level for all tests was set at *p* < 0.05. Given the exploratory nature of this study, no adjustment for multiple testing was performed.

## Results

### Participants

**Fig. 1:**
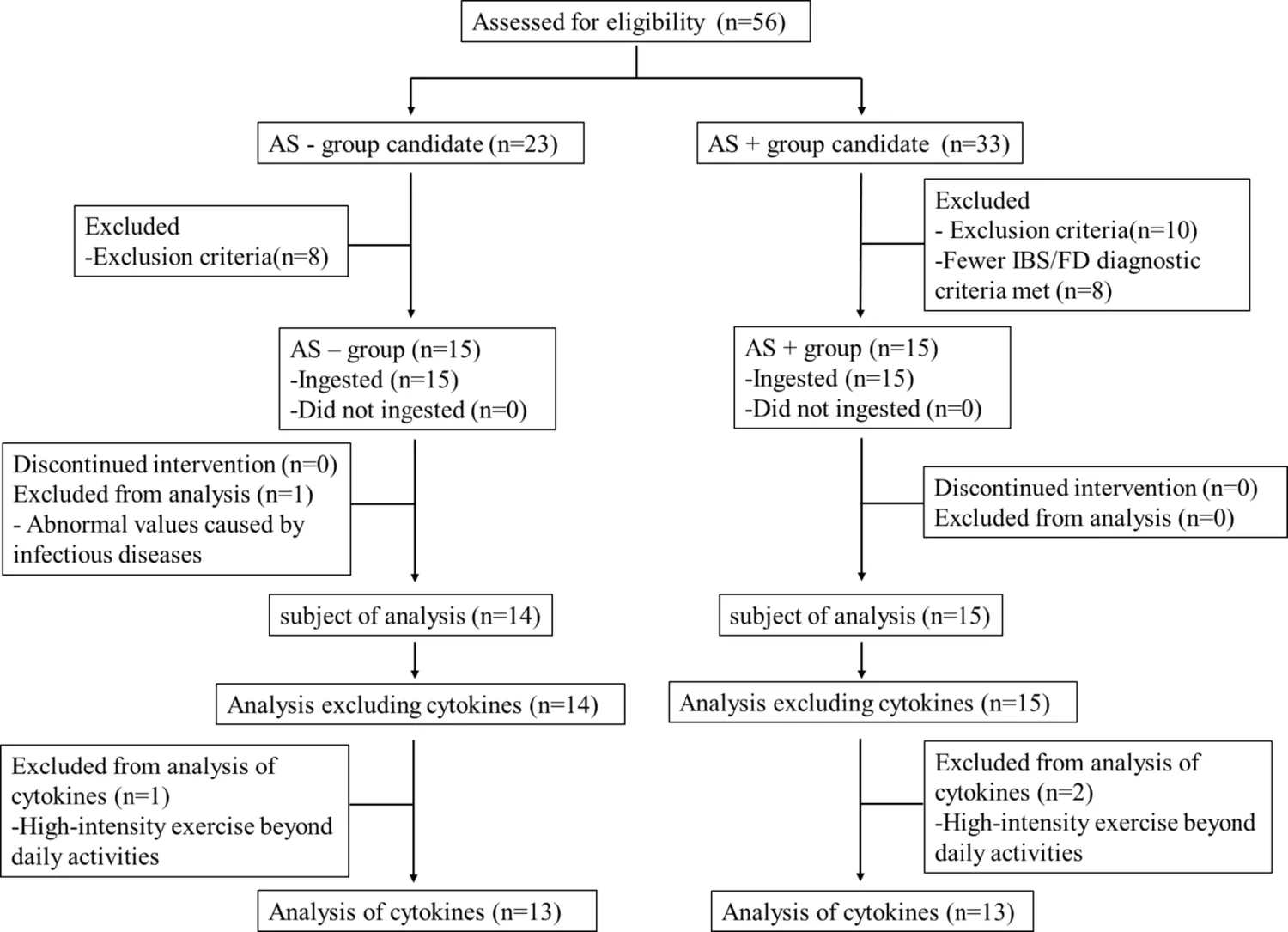
Study flowchart. AS+, Abdominal symptom-positive group; AS−, Abdominal symptom-negative group

Table 2 presents the characteristics of the study participants, and Fig. 1 illustrates the flow of participant allocation by group. Analysis of dietary records throughout the intervention revealed that, for all participants, meals containing wheat-based processed foods accounted for < 20% of the total number of recorded meals; therefore, no participants met the predefined exclusion criteria. One participant in the AS− group was excluded from the analysis after week 4 assessments revealed increased IFN-γ and IL-6 levels accompanied by cold-like symptoms, indicating an intercurrent infection. Three participants reporting habitual physical activity levels beyond typical daily intensity, potentially influencing inflammatory cytokine profiles, were excluded from the cytokine analyses, resulting in a final cytokine analysis population of 26 participants. Based on dietary records, we estimated each participant’s yellow pea intake from the yellow pea content of the yellow pea–based products (YPP, YPB, and YPC). Subgroup analyses were then conducted for participants who consumed ≥ 80 g/day of yellow peas (*n* = 27) and for participants whose intake of wheat-based processed foods accounted for ≤ 10% of their total dietary intake. This percentage was calculated on a meal basis; that is, wheat-based processed foods were considered present if they appeared in a meal, and the proportion was defined as the number of meals containing such foods divided by the total number of meals.

**Table 2:** Baseline characteristics of study participants

	AS− (*n* = 14)		AS+ (*n* = 15)		*p*^*2*^
Age (years)	46.7	(10.79)	39.4	(12.44)	0.099
Men (*n*)	5		6		
Body Weight(kg)	60.5	(9.01)	58.5	(8.00)	0.530
BMI (kg/ m^2^)	21.4	(2.00)	21.1	(1.66)	0.609
SBP (mmHg)	117.0	(17.03)	108.7	(9.46)	0.224
DBP (mmHg)	68.0	(10.31)	67.6	(8.64)	0.911
Fulfilling both IBS and FD criteria^1^ (*n*, %)	0 (0.0)		8 (53.3)		
Fulfilling FD criteria^1^ (*n*, %)	0 (0.0)		6 (40.0)		
Fulfilling IBS criteria^1^ (*n*, %)	0 (0.0)		1 (6.7)		
Abdominal pain or discomfort	0.5	(1.61)	6.2	(1.42)	0.000
Nausea and vomiting	0.2	(0.8)	0.9	(1.58)	0.154
Abdominal distension	0.9	(1.59)	5.9	(2.42)	0.000
Decreased passage of stools	2.8	(3.49)	3.2	(2.62)	0.435
Increased passage of stools	2.4	(3.34)	3.1	(3.39)	0.514
Loose stools	1.4	(2.21)	4.2	(2.24)	0.002
Hard stools	1.4	(1.95)	4.1	(2.85)	0.007
Urgent need for defecation	0.6	(1.6)	3.0	(2.67)	0.004
Feeling of incomplete	1.5	(1.99)	3.6	(3.29)	0.041
Dermatitis	0.5	(0.94)	0.6	(1.12)	1.000
Headache	0.2	(0.43)	1.5	(2.)	0.125
Foggy mind	0.3	(0.61)	1.7	(2.47)	0.158
Fatigue	2.4	(2.59)	3.8	(2.11)	0.057
Joint muscle pains	0.9	(1.96)	0.7	(1.83)	0.882

Data are presented as the means (SD). ^1^According to the Rome IV criteria. ^2^ For normally distributed data, between-group comparisons were performed using an unpaired t-test, whereas for non-normally distributed data, the Wilcoxon rank-sum test was used. AS+, Abdominal symptom-positive group; AS−, Abdominal symptom-negative group; BMI, Body mass index; SBP, Systolic blood pressure; DBP, Diastolic blood pressure.

Supplementary Table S1 presents the vital signs and body composition measurements of the participants. Safety was evaluated through physician interviews and adverse event assessments. No intervention-related adverse events were observed, and no participants reported the use of medications that could potentially influence the study outcomes during the intervention period.

### Effect of reduced gluten intake on clinical symptoms

Supplementary Table 2 presents the results of clinical symptom evaluations, including abdominal symptoms, at weeks 0, 2, and 4 in the AS− group and AS+ group. At baseline (week 0), the AS+ group exhibited significantly higher scores than those of the AS− group in six symptoms: abdominal pain or discomfort (*p* < 0.001), abdominal distension (*p* < 0.001), loose stools (*p* = 0.039), hard stools (p = 0.003), urgent need for defecation (*p* = 0.007), and feeling of incomplete evacuation (*p* = 0.007). In contrast, no significant between-group differences were observed for nausea or vomiting, decreased passage of stools, increased passage of stools, dermatitis, headache, foggy mind, fatigue or joint/muscle pain.

**Fig. 2:**
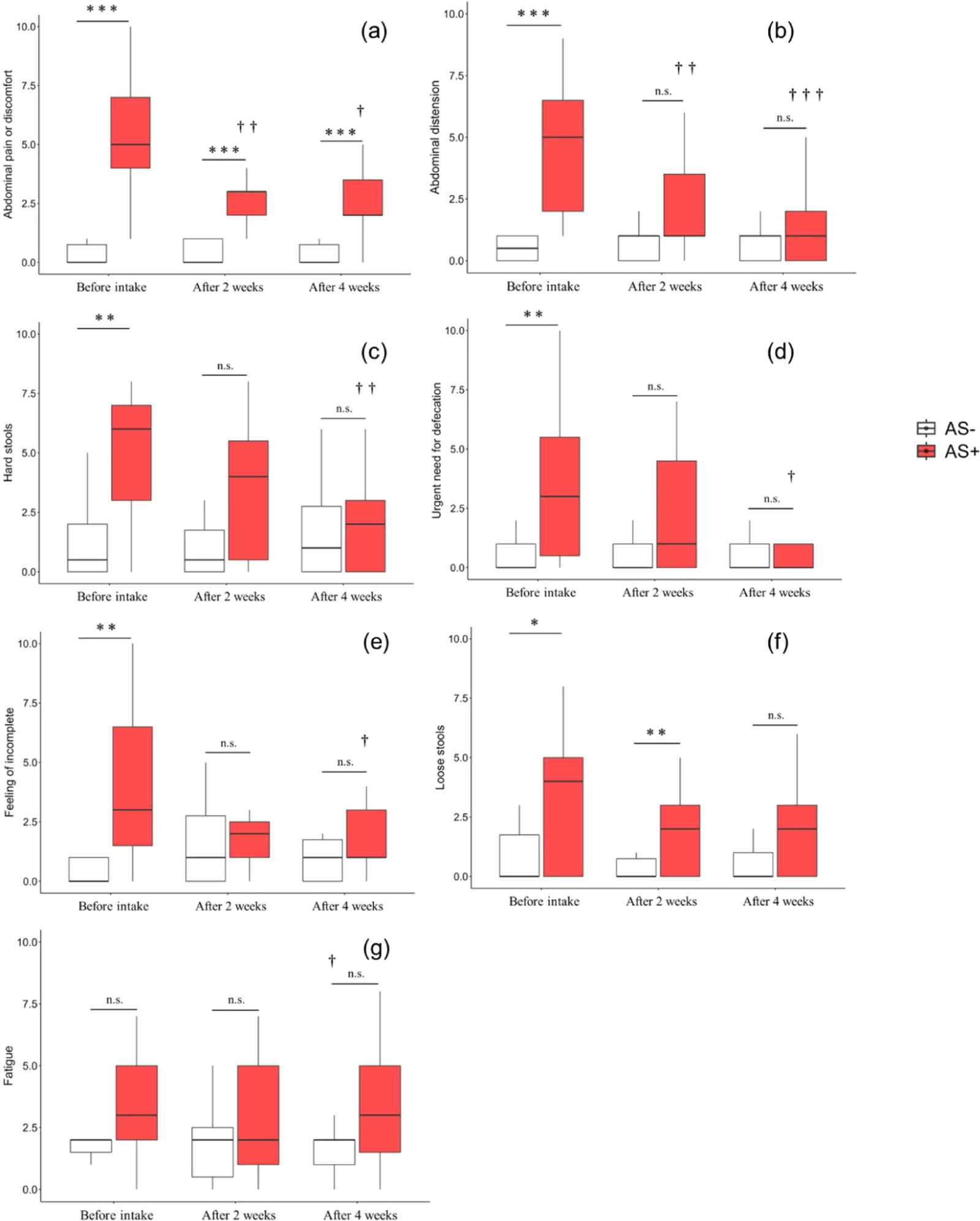
Effect of reduced gluten intake on clinical symptom improvement. Data are presented as medians (interquartile ranges). Significant differences between groups are indicated by *, and significant within-group differences from before intake are indicated by †. * *p* < 0.05, ** *p* < 0.01, *** *p* < 0.001. ^†^ *p* < 0.05, ^††^ *p* < 0.01, ^†††^ *p* < 0.001. n.s., not significant (no significant difference between groups)

Following gluten intake reduction, the AS+ group exhibited significant improvements in abdominal pain or discomfort at weeks 2 and 4 (*p* = 0.003 and *p* = 0.021, respectively) and in abdominal distension at weeks 2 and 4 (*p* = 0.002 and *p* < 0.001, respectively), while hard stools (*p* = 0.002), urgent need for defecation (*p* = 0.011), and feeling of incomplete evacuation (*p* = 0.043) showed significant improvement at week 4. From week 2 onwards, scores for abdominal distension, hard stools, urgent need for defecation, and feeling of incomplete evacuation in the AS+ group decreased, after which the between-group differences were no longer significant (Fig. 2a–e). For loose stools, the AS+ group score decreased at week 4, eliminating the previously observed between-group differences (Fig. 2f). In contrast, for parameters that exhibited no between-group differences at baseline, including nausea or vomiting, decreased and increased passage of stools, dermatitis, headache, foggy mind, fatigue or joint/muscle pain, neither within-group changes nor between-group differences reached statistical significance after the intervention.

To further explore the association between intake of the test food products and clinical symptoms, the intake of yellow peas was calculated from dietary records.. A subgroup analysis of the clinical symptom questionnaire restricted to participants with an average daily intake of 80 g or more was performed. A significant improvement in fatigue was observed in the AS− group (*p* = 0.016) (Fig. 2g).

### Effect of reduced gluten intake on QOL

Table 3 presents the findings from the QOL questionnaire for the AS− and AS+ groups at baseline (week 0) and week 4. At baseline, the AS+ group exhibited significantly lower scores than those of the AS− group across four domains: the MCS (*p* = 0.006), general health (*p* = 0.008), social functioning (*p* = 0.007), and mental health (*p* = 0.004), indicating diminished QOL. No significant between-group differences were observed in the remaining domains.

**Table 3:** Effect of reduced gluten intake on quality of life

		Week 0		*p*^1^	Week 4		*p*^2^	*p*^3^
PCS	AS−	54.53	(52.12, 56.94)	0.495	55.76	(53.87, 57.64)	0.068	0.082
	AS+	53.02	(49.01, 57.03)		52.61	(49.63, 55.60)		0.697
MCS	AS−	58.98	(54.88, 63.08)	0.006**	58.72	(55.40, 62.04)	0.076	0.854
	AS+	50.49	(45.98, 54.99)		52.96	(47.20, 58.72)		0.248
RCS	AS−	51.75	(49.46, 54.04)	0.666	50.39	(48.73, 52.04)	0.915	0.138
	AS+	50.75	(46.40, 55.10)		50.61	(46.40, 54.82)		0.939
Physical functioning	AS−	96.79	(94.36, 99.22)	0.551	96.79	(94.96, 98.61)	0.323	1.000
	AS+	95.00	(91.53, 98.47)		94.67	(91.81, 97.53)		1.000
Role physical	AS−	96.44	(89.70, 103.17)	0.077	97.33	(93.41, 101.25)	0.083	1.000
	AS+	90.42	(84.17, 96.67)		90.43	(82.81, 98.04)		1.000
Bodily pain	AS−	89.93	(81.47, 98.39)	0.072	89.57	(82.35, 96.79)	0.068	1.000
	AS+	76.27	(64.96, 87.58)		77.40	(67.23, 87.57)		0.723
General health	AS−	78.14	(70.72, 85.57)	0.008**	78.79	(71.48, 86.09)	0.056	0.685
	AS+	61.80	(52.20, 71.40)		66.13	(54.72, 77.55)		0.217
Vitality	AS−	69.66	(59.67, 79.66)	0.104	71.44	(62.09, 80.80)	0.173	0.515
	AS+	58.77	(49.09, 68.46)		62.52	(52.47, 72.57)		0.394
Social functioning	AS−	99.11	(97.18, 101.04)	0.007**	91.96	(83.66, 100.27)	0.797	0.063
	AS+	84.17	(73.55, 94.78)		90.83	(83.22, 98.45)		0.188
Role emotional	AS−	93.46	(87.15, 99.76)	0.386	94.65	(89.45, 99.85)	0.330	0.500
	AS+	87.78	(79.26, 96.30)		86.11	(75.27, 96.96)		0.703
Mental health	AS−	84.64	(77.90, 91.39)	0.004**	83.21	(77.71, 88.72)	0.061	0.686
	AS+	70.67	(63.74, 77.60)		72.67	(62.64, 82.70)		0.526

Data are presented as means (95% confidence intervals). ^1,2^ For normally distributed data, between-group comparisons were performed using an unpaired t-test, whereas for non-normally distributed data, the Wilcoxon rank-sum test was used. ^3^ For normally distributed data, within-group comparisons (week 0 vs. week 4) were performed using a paired t-test, whereas for non-normally distributed data, the Wilcoxon signed-rank test was used. * *p* < 0.05, ** *p* < 0.01. PCS, Physical component summary; MCS, Mental component summary; RCS, Role/Social component summary.

No significant differences were detected across any domain when comparing pre- and post-intervention measurements. By week 4, the between-group differences observed at baseline in the MCS, general health, social functioning, and mental health, along with other domains, disappeared. In addition, a subgroup analysis was conducted excluding participants whose intake of wheat-based foods accounted for 10% or more of total eating occasions, calculated on a meal basis as defined in the methods. In this analysis, a stricter cutoff (10%) than the predefined adherence criterion (20%) was applied. As a result, a significant improvement in the PCS was observed within the AS− group (*p* = 0.042) (Supplementary Table 3).

### Biochemical parameters

Table 4 presents the results for biological parameters at week 0 and week 4 in the AS− and AS+ groups. No significant intergroup differences were observed between the AS− and AS+ groups at both time points.

**Table 4:** Effect of reduced gluten intake on biochemical parameters

		Week 0		*p*^1^	Week 4		*p*^2^	*p*^3^
IFN-γ (fg/mL)	AS−	541.5	(419.33, 663.60)	0.579	752.8	(433.13, 1072.57)	0.614	0.273
	AS+	918.9	(210.86, 1626.99)		814.8	(322.03, 1307.66)		0.642
IL-1β (fg/mL)	AS−	27.1	(10.97, 43.14)	0.135	296.6	(− 272.49, 865.71)	0.390	0.176
	AS+	41.8	(18.95, 64.55)		189.3	(− 30.59, 409.14)		0.455
IL-2 (fg/mL)	AS−	172.5	(75.66, 269.32)	0.104	182.4	(80.62, 284.24)	0.579	0.248
	AS+	125.0	(79.91, 170.12)		173.9	(94.74, 252.96)		0.021*
IL-4 (fg/mL)	AS−	39.0	(4.64, 73.35)	0.441	37.9	(3.04, 72.68)	0.687	0.428
	AS+	22.7	(11.28, 34.04)		25.0	(12.06, 37.90)		0.946
IL-6 (fg/mL)	AS−	1794.4	(749.71, 2839.06)	0.772	1190.0	(894.49, 1485.51)	0.190	0.893
	AS+	1155.5	(901.08, 1409.84)		1967.2	(912.54, 3021.92)		0.040*
IL-10 (fg/mL)	AS−	626.2	(305.79, 946.67)	0.650	542.4	(389.64, 695.07)	0.762	0.542
	AS+	532.8	(310.90, 754.79)		760.9	(393.57, 1128.27)		0.110
IL-12p70 (fg/mL)	AS−	472.0	(201.93, 742.16)	0.869	481.1	(219.80, 742.35)	0.960	0.831
	AS+	367.9	(279.27, 456.58)		368.4	(286.46, 450.31)		0.992
IL-17A (fg/mL)	AS−	389.8	(274.00, 505.54)	0.880	418.3	(291.31, 545.30)	0.614	0.677
	AS+	408.4	(235.73, 581.04)		483.8	(328.30, 639.24)		0.309
TNF-α (fg/mL)	AS−	269.7	(203.82, 335.57)	0.696	284.3	(217.41, 351.20)	0.479	0.123
	AS+	241.4	(207.02, 275.75)		293.8	(233.28, 354.41)		0.050
IL-13 (fg/mL)	AS−	22.4	(13.71, 31.07)	0.441	31.4	(17.99, 44.80)	0.067	0.110
	AS+	18.6	(12.90, 24.37)		18.3	(12.44, 24.08)		0.892
Human Anti-Gliadin IgG (U/mL)	AS−	23.5	(13.43, 33.57)	0.652	23.2	(13.61, 32.83)	0.591	0.662
	AS+	29.0	(14.25, 43.69)		28.7	(15.05, 42.43)		0.978
Fe (µg/L)	AS−	1000.7	(810.00, 116.00)	0.415	1092.9	(720.00, 1470.00)	0.195	0.201
	AS+	1113.3	(845.00, 1332.50)		919.3	(610.00, 1150.50)		0.169
Zn (µg/L)	AS−	874.3	(815.00, 925.00)	0.954	895.7	(870.00, 917.50)	0.189	0.263
	AS+	872.0	(780.00, 950.00)		853.3	(815.00, 900.00)		0.495
Ferritin (ng/mL)	AS−	88.9	(25.37, 152.84)	0.394	88.1	(24.76, 151.38)	0.644	0.756
	AS+	63.4	(23.36, 102.83)		71.1	(28.59, 113.63)		0.890

Data are presented as means (95% confidence intervals). ^1,2^ For normally distributed data, between-group comparisons were performed using an unpaired t-test, whereas for non-normally distributed data, the Wilcoxon rank-sum test was applied. ^3^ For normally distributed data, within-group comparisons (week 0 vs. week 4) were performed using a paired t-test, whereas for non-normally distributed data, the Wilcoxon signed-rank test was applied. * *p* < 0.05. IFN-γ, interferon-γ; IL, interleukin; TNF-α, tumour necrosis factor-α; IgG, immunoglobulin G; Fe, iron; Zn, zinc.

In contrast, in the AS+ group, a significant increase in serum IL-2 and IL-6 levels was observed following the intervention. However, IL-6 concentrations remained below 5000 fg/mL for nearly all participants regardless of measurement time point, except for one participant in the AS− group at week 0 and one in the AS+ group at week 4, and the median IL-6 value in each group was approximately 1000 fg/mL, indicating that serum IL-6 concentrations in the study participants remained within normal limits. No significant differences were observed in other assessed parameters.

### Gut microbiota analysis

**Fig. 3:**
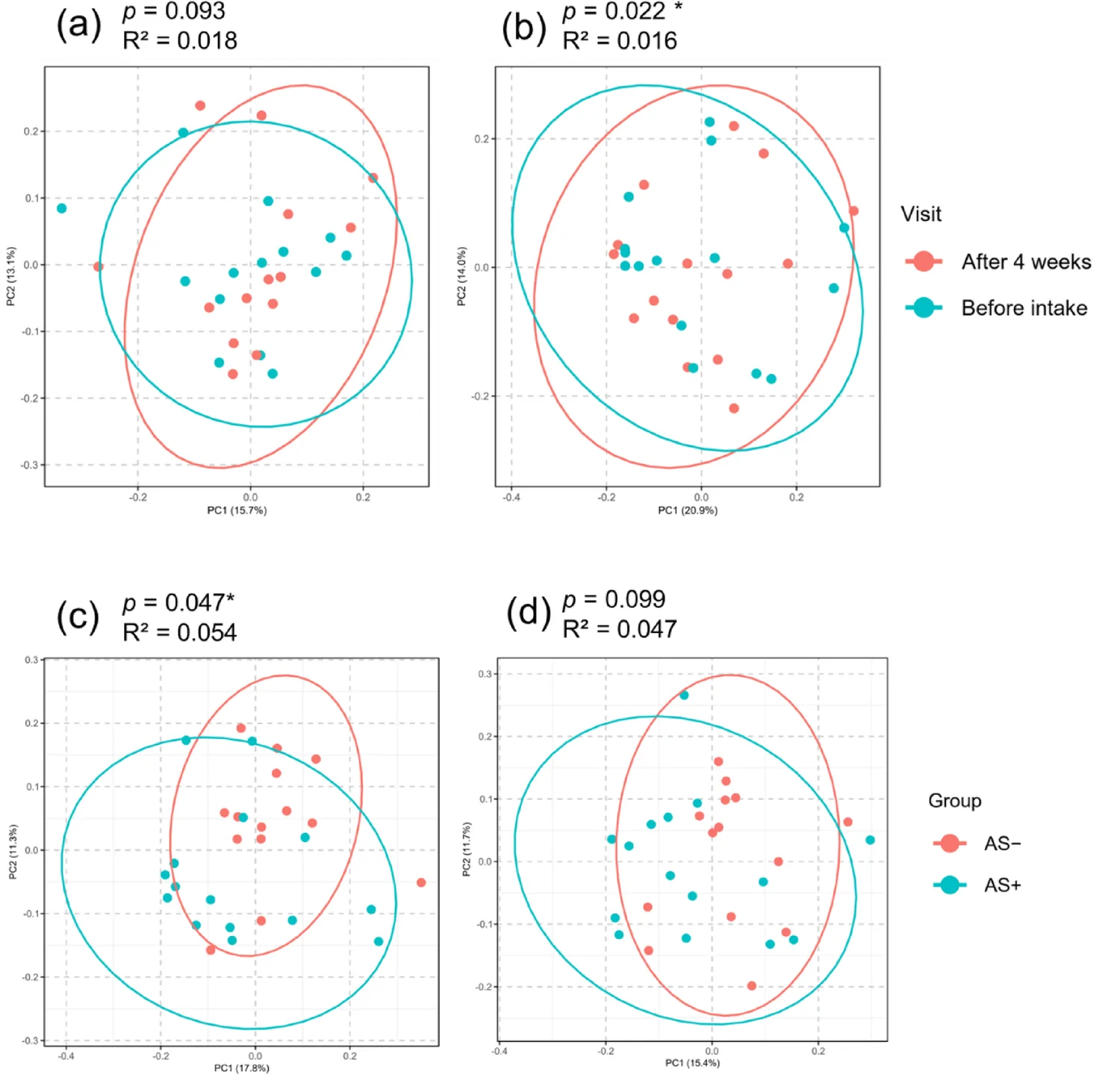
Effect of reduced gluten intake on β-diversity. **a** Within-group comparison in the AS− group. **b** Within-group comparison in the AS+ group. **c** Between-group comparison before intervention. **d** Between-group comparison after intervention. * *p* < 0.05

Alpha diversity revealed no significant between-group or pre- versus post-intervention differences (Supplementary Table 4). β-diversity analysis showed no significant differences in the pre- and post-intervention comparison in the AS− group (Fig. 3a), whereas significant differences were observed in the AS+ group (Fig. 3b). Additionally, significant differences were detected in the between-group comparison at baseline (week 0), which disappeared by week 4 (Fig. 3c, d).

Changes in the relative abundance of the gut microbiota were evaluated following the intervention (Supplementary Table 5). At the family level, the relative abundance of *Oscillospiraceae* decreased significantly in the AS− group (*p* = 0.030). At the genus level, *Lachnospiraceae_NK4A136_group; unclassified* and *Negativibacillus; unclassified* were significantly increased in the AS− group (*p* = 0.014 and *p* = 0.047, respectively).

Between-group comparisons of the magnitude of change in relative abundance revealed significant differences at the family level for *Oscillospiraceae, Eggerthellaceae; unclassified*, *Muribaculaceae; unclassified*, *Lachnospiraceae; unclassified*, *Ruminococcaceae; unclassified*, and *Lachnospiraceae; unclassified; human.gut.metagenome* (*p* = 0.019, 0.016, 0.042, 0.017, 0.049, and 0.035, respectively). At the genus level, significant differences were observed in *Negativibacillus; unclassified, Butyricimonas; unclassified*, *Holdemanella, Defluviitaleaceae UCG011; unclassified*, *Eisenbergiella; unclassified*, *Eubacterium hallii group*, *Eubacterium hallii group; unclassified*, *Oscillospiraceae UCG005*, *Incertae Sedis*, *Phocea; unclassified*, and *Megasphaera* (*p* = 0.047, 0.015, 0.032, 0.017, 0.030, 0.047, 0.019, 0.015, 0.023, 0.019, and 0.025, respectively). At the species level, *Bacteroides dorei* differed significantly between groups (*p* = 0.021) (Supplementary Table 5).

### Correlation analysis between gut microbiota and clinical symptoms

**Fig. 4:**
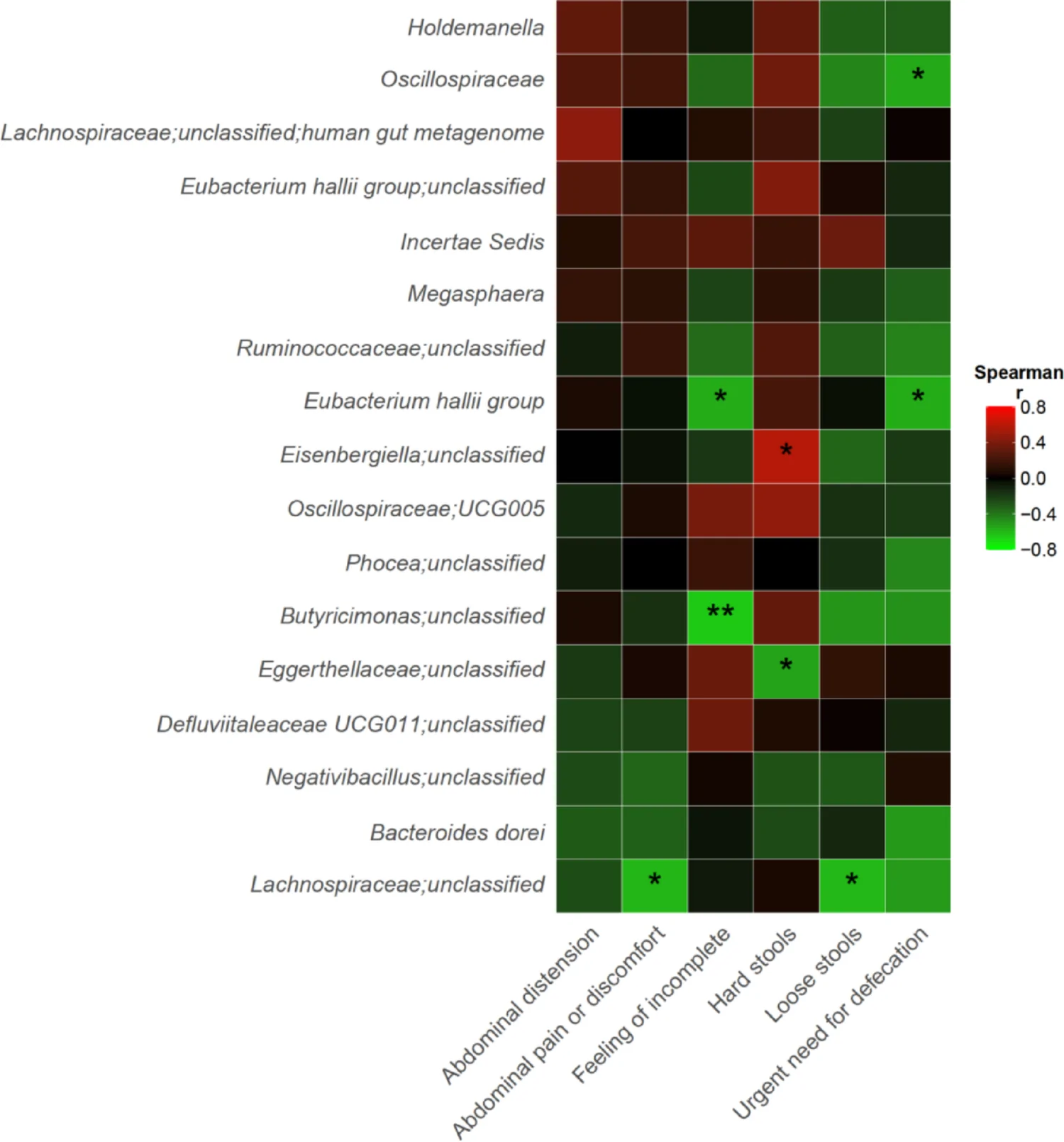
Spearman correlation between changes in gut microbiota and changes in clinical symptom scores. Spearman’s correlation coefficients (r) between changes in abdominal symptoms and gut microbiota in the AS+ group are presented as a heatmap. Red denotes positive correlations, whereas green denotes negative correlations. * *p* < 0.05, ** *p* < 0.01

Spearman’s correlation analysis examined the association between changes in gut microbiota composition and changes in clinical symptom scores in the AS+ group (Fig. 4). At the family level, an increase in *Lachnospiraceae; unclassified* exhibited a significant negative correlation with improvements in abdominal pain or discomfort and loose stools (*p* = 0.021, *p* = 0.019), while an increase in *Oscillospiraceae* exhibited a significant negative correlation with improvement in urgent need for defecation (*p* = 0.033). Furthermore, an increase in the *Eggerthellaceae group* exhibited a significant negative correlation with improvement in hard stools (*p* = 0.040).

At the genus level, an increase in *Butyricimonas; unclassified* exhibited a significant negative correlation with improvement in feeling of incomplete evacuation (*p* = 0.009), and an increase in the *Eubacterium hallii group* exhibited a significant negative correlation with improvements in both urgent need for defecation and feeling of incomplete evacuation (*p* = 0.030, *p* = 0.030). In contrast, the change in *Eisenbergiella; unclassified*, exhibited a significant positive correlation with changes in the hard stools score (*p* = 0.022), and subjects who exhibited a decrease in this genus exhibited symptomatic improvement.

## Discussion

In this study, participants with high habitual wheat consumption, with or without self-reported abdominal symptoms, underwent a reduced gluten intake intervention using YPP, YPB, and YPC. This intervention was associated with improvements in abdominal symptoms and QOL. Notably, in the AS+ group (meeting the Rome IV diagnostic criteria for IBS and/or FD), β-diversity analysis demonstrated significant differences in gut microbiota composition between pre- and post-intervention assessments. Based on these pre- and post-intervention changes, exploratory correlation analyses were performed to investigate potential associations between alterations in gut microbiota and changes in abdominal symptoms. Significant correlations were observed between symptom improvement and changes in the relative abundance of specific gut bacterial taxa. These observations are consistent with previous reports suggesting that reduced gluten intake is associated with alleviation of abdominal symptoms in individuals with NCGS [4]. Taken together, our study suggests that YPP, YPB, and YPC may serve as novel dietary options for gluten reduction in individuals with wheat-related abdominal symptoms. However, given the present study design, causal relationships and the underlying mechanisms linking dietary changes, gut microbiota alterations, and symptom improvement could not be fully elucidated. In addition, the observed benefits cannot be attributed solely to gluten reduction and may also be explained by increased dietary fiber intake, changes in fermentable substrates, or broader modifications in overall dietary composition, or placebo effects.

Several bacterial taxa associated with maintaining gut and anti-inflammatory effects were identified among those correlated with symptom improvement. For instance, the *Eubacterium hallii group*, which exhibited significant correlations with improvements in urgent bowel movements and the feeling of incomplete evacuation, produces butyrate and propionate and has been reported to be significantly decreased in patients with inflammatory bowel disease (IBD), Crohn's disease (CD), and ulcerative colitis (UC) compared to healthy individuals [25]. Similarly, *Butyricimonas*, which exhibited a significant correlation with improvement in the feeling of incomplete evacuation, also produces butyrate and has been reported to be decreased in patients with UC [26]. These observations suggest a potential association between an increase in butyrate-producing gut bacteria and a reduction in abdominal symptoms, possibly mediated by improvements in intestinal homeostasis and barrier function, though this mechanistic interpretation remains speculative given the exploratory nature of the study. Furthermore, previous studies have reported that butyrate possesses anti-carcinogenic properties [27], and that constipation is associated with an increased risk of colorectal cancer [28]. In the AS+ group, an increase in butyrate-producing gut bacteria was associated with improvements in bowel movement symptoms, such as hard stools and the sensation of incomplete evacuation. Together, these findings raise the possibility that improvements in bowel movement symptoms accompanied by increased abundance of butyrate-producing bacteria may be related to broader colorectal health outcomes. However, given the exploratory design, small sample size, and short intervention duration (4 weeks), this possibility cannot be addressed by the present study and requires confirmation in larger, adequately powered longitudinal trials.

In this study, no significant changes were observed in clinical symptoms, QOL, or cytokine markers in the AS− group; however, a significant increase in *Lachnospiraceae_NK4A136_group* was detected in the gut microbiota. This SCFA-producing bacterium was negatively correlated with intestinal permeability and plasma lipopolysaccharide (LPS) levels, while positively correlated with colon length and claudin-1 (Cldn1) expression in diet-induced obese mice [29]. The disruption of the intestinal barrier function leads to a leaky gut condition, in which bacterial-derived components, including LPS and endotoxins, leak into the systemic circulation, which is closely associated with metabolic and autoimmune diseases, including obesity, neurodegenerative disorders, cardiovascular disease, IBD, and type 1 diabetes mellitus [30, 31]. Therefore, although the changes in gut microbiota in the AS− group were not accompanied by short-term improvements in subjective symptoms or clinical indicators, they may have potential implications for intestinal barrier function and systemic health, particularly over the medium to long term. However, these possibilities remain speculative and warrant further investigation in future longitudinal studies. ​

GFD has been suggested to result in a deficiency of prebiotic fibres due to the elimination of wheat products, thereby decreasing SCFA-producing bacteria that are typically beneficial in healthy individuals. When healthy individuals followed a GFD for 4 weeks, significant decreases in the SCFA-producing bacteria, including *Ruminococcus bromii*, *Roseburia faecis*, and *Bifidobacterium longum*, were observed compared to those consuming a regular diet [13, 14]. A low-gluten diet for 8 weeks (approximately 2 g/day) resulted in lower levels of butyrate-producing bacteria, including *Anaerostipes hadrus* and *Eubacterium hallii* [32], than those associated with a high-gluten diet (approximately 18 g/day). However, in the present study, no reduction in SCFA-producing bacteria was observed in the AS+ group, and an increase in *Lachnospiraceae_NK4A136_group; unclassified*, an SCFA-producing bacterium, was observed in the AS− group, indicating that these beneficial bacteria were maintained or increased despite gluten intake reduction through the inclusion of yellow pea–based products. This may be attributed to the dietary fibre and resistant starch derived from yellow peas, which likely served as substrates for the gut microbiota. ​

Yamada et al. previously reported that consuming 80 g of YPP composed solely of yellow peas once daily for 4 weeks increased SCFA-producing bacteria, such as *Bifidobacterium longum* and *Ruminococcus bromii* [17], which aligns with the findings of our study. Therefore, when implementing a GFD, incorporating yellow peas as primary ingredients may help maintain gut microbiota composition and could be associated with improvements in abdominal symptoms and QOL, suggesting that such an approach may represent a promising alternative to conventional GFD strategies.

In the present study, improvements were observed in both hard stool and loose stool symptoms following the intervention. Yellow pea products are rich in dietary fiber, which may have contributed to these changes. Dietary fiber has been reported to mechanically stimulate the colonic mucosa, promote intestinal motility and intestinal fluid secretion, increase stool bulk, and accelerate colonic transit in individuals with hard stools or constipation. Conversely, dietary fiber can also absorb and retain excess luminal water, thereby improving stool consistency and normalizing stool form in individuals with loose stools or diarrhea-like symptoms [33]. Therefore, the dietary fiber contained in yellow peas may have contributed to the improvement of both hard and loose stool symptoms observed in this study. However, given the small sample size and exploratory nature of the study, these findings should be interpreted with caution, and the observed associations should not be interpreted as evidence of causality.

In the present study, abdominal symptoms, including abdominal distension, were significantly improved in the AS+ group following the intervention. FODMAP intake is known to induce gas-related symptoms in susceptible individuals. Nevertheless, abdominal distension significantly improved in the AS+ group in the present study. Previous reports have shown that the FODMAP content of yellow pea and wheat flour does not differ substantially; therefore, changes in FODMAP intake resulting from the intervention were likely limited [7, 8]. However, the intervention likely resulted in a shift in the predominant FODMAP species consumed, from wheat-derived fructans to yellow pea-derived GOS. Therefore, a potential contribution of qualitative differences in FODMAP composition to the observed symptom changes cannot be ruled out. However, dietary intake in this study was assessed using self-reported records, and actual FODMAP intake could not be quantified accurately. In addition, the sample size was relatively limited. Therefore, these interpretations should be made with caution. Further studies with larger sample sizes and more precise assessments of FODMAP intake are warranted to clarify the mechanisms underlying the observed changes in gastrointestinal symptoms.

NCGS has been reported to overlap with IBS and/or FD. However, due to the challenges associated with its diagnosis, many individuals may unknowingly continue to consume gluten. In the present study, we targeted individuals who exhibited abdominal symptoms despite habitual high wheat consumption. Consequently, our analyses were based on a mixed phenotype, which may have obscured gut microbiota characteristics specific to individuals presenting exclusively with IBS or FD symptoms. Future studies focusing on individuals with habitual high wheat consumption who exhibit only IBS or only FD symptoms may provide further insights into disease-specific microbial profiles.

In the AS+ group, a significant increase in serum IL-2 and IL-6 levels was observed following the intervention. IL-2 is an essential cytokine for the differentiation and maintenance of anti-inflammatory regulatory T cells [34]. IL-2 has been reported to contribute to the maintenance of immune homeostasis. In a murine model of UC, low-dose IL-2 administration improved clinical symptoms and increased goblet cell numbers and mucin expression, enhancing intestinal mucus layer formation. The expression of tight junction-associated molecules, including zonula occludens-1, claudin-1, and E-cadherin, was re-established, supporting functional restoration of the intestinal epithelial barrier. Increased expression of these barrier molecules has been associated with intestinal epithelial apoptosis suppression in mice with dextran sulphate sodium-induced colitis, indicating that IL-2 may also contribute to the maintenance of structural homeostasis in the intestinal mucosa [35]. Clinical trials in patients with UC have reported that subcutaneous low-dose IL-2 resulted in clinical improvement in 53% of patients and clinical remission in 21%, indicating that IL-2 may also exert anti-inflammatory effects in humans [36]. Taken together, these findings suggest that the observed increase in IL-2 levels in the present study may be associated with physiological immune modulation related to intestinal barrier function; however, the clinical significance of this change warrants further investigation.

IL-6 is typically characterised as a pro-inflammatory cytokine; however, it exhibits context-dependent immunomodulatory properties and can also mediate anti-inflammatory responses [37]. Consistent with this dual function, in a combined pre- and probiotic intervention study in patients with IBS, elevated serum IL-6 levels paralleled improvements in abdominal symptoms and QOL, indicating that IL-6 may be involved in the maintenance of homeostasis [38]. Although a significant increase in IL-6 levels was observed after the intervention, the overall values remained within the estimated physiological range for healthy individuals (5186 fg/mL) [39]. Notably, only one participant exhibited a post-intervention IL-6 concentration slightly above 5000 fg/mL, and the median level remained close to 1000 fg/mL. Therefore, this degree of variation was not considered indicative of abnormal IL-6 elevation. Furthermore, IL-6 is also implicated in intestinal barrier function and epithelial repair. Under physiological conditions, intraepithelial lymphocytes secrete IL-6 in response to stimulation by the intestinal microbiota and have been reported to contribute to the regulation of intestinal permeability and the maintenance of mucin production [40]. Collectively, these findings suggest that the increase in IL-6 levels observed in the present study may represent a physiological response rather than a pathological inflammatory process, as these values remained within the normal physiological range and did not indicate systemic inflammation.

Based on dietary diary data, yellow pea consumption from YPP, YPB, and YPC was calculated, and a stratified analysis revealed that participants with an average intake of ≥ 80 g/day showed a significant reduction in fatigue within the AS− group. The antioxidant properties of yellow pea may be implicated as potential mechanisms underlying fatigue reduction. Itoh et al. reported that healthy adult men and women (*n* = 40) who consumed 80 g of yellow peas once daily for four weeks exhibited significant improvements in oxidative stress markers compared to the control group, and discussed that the polyphenols contained in yellow peas contributed to these antioxidant effects [24]. A close relationship between oxidative stress and fatigue was previously reported; furthermore, in a trial administering aged garlic extract for 12 weeks to healthy participants, serum glutathione peroxidase, reflecting the antioxidant capacity, was increased, while urinary isoprostane concentration, reflecting in vivo lipid oxidation, was decreased [41]. Concurrently, subjective fatigue was significantly improved [42]. Taken together, these findings suggest that attenuation of oxidative stress may underlie the reduction in fatigue observed in the present study.

This study had some limitations. First, given that this was an exploratory study and the sample size was not determined based on statistical power considerations, the findings should be interpreted with caution, particularly regarding changes in gut microbiota composition and cytokine levels. In addition, potential selection bias due to recruitment via a commercial research platform and the relatively healthy, culturally specific study population may limit the external validity of the findings. Second, dietary records were self-reported, and the possibility of reporting bias, including under- or over-reporting of food intake, cannot be excluded. Second, dietary records were self-reported, and the possibility of reporting bias, including under- or over-reporting of food intake, cannot be excluded. Similarly, the exclusion of coeliac disease and other organic gastrointestinal disorders relied on self-reported medical history rather than systematic clinical evaluation or validated biomarker testing, and therefore misclassification bias cannot be excluded. In addition, accurate quantitative estimates of gluten intake and wheat-derived fermentable carbohydrates could not be derived from the dietary records. Therefore, the extent to which gluten intake or related dietary components were reduced during the intervention period could not be precisely determined. Third, this study involved concurrent reduced gluten intake with the introduction of YPP, YPB, and YPC as intervention foods; it was not possible to clearly distinguish whether the observed effects were attributable to gluten elimination, the constituent components of these products, quantitative or qualitative changes in FODMAP exposure, potential placebo effects, or their synergistic effects. In particular, placebo response rates have been reported to be relatively high in dietary intervention studies targeting gastrointestinal symptoms [43]. Therefore, the contribution of placebo effects to the observed improvements cannot be excluded. Future investigations should adopt multi-group comparative trials to individually validate each factor. Fourth, this trial was funded by Mizkan Holdings Co., Ltd., which may introduce potential bias despite predefined protocols and independent data analysis; therefore, the influence of industry funding on study design, data interpretation, and reporting cannot be completely excluded. Finally, although associations between changes in gut microbiota composition and symptom improvement were observed, further studies are needed to better elucidate the nature of these relationships.

In conclusion, among individuals experiencing wheat-associated abdominal symptoms, a dietary replacement strategy using YPP, YPB, and YPC was associated with improvements in abdominal symptoms and QOL, without marked disruption of gut microbiota composition. These findings suggest that, at least under the conditions examined in this study, YPP, YPB, and YPC may have potential as functional foods for gluten reduction.

## Supplementary Information

Below is the link to the electronic supplementary material.

Supplementary Material 1

## Data Availability

The dataset analysed in this study is not publicly available because of the lack of participant consent for data sharing and confidentiality restrictions; however, it is available from the corresponding author upon reasonable request.

## Abbreviations

AS+ group: Abdominal symptom-positive group
AS− group: Abdominal symptom-negative group
CD: Crohn’s disease
FD: Functional dyspepsia
GFD: Gluten-free diet
IBD: Inflammatory bowel disease
IBS: Irritable bowel syndrome
LPS: Lipopolysaccharide
MCS: Mental component summary
NCGS: Non-coeliac gluten sensitivity
PCS: Physical component summary
QOL: Quality of life
RCS: Role/social component summary
SCFA: Short-chain fatty acid
TREND: Transparent reporting of evaluations with nonrandomized designs
UC: Ulcerative colitis
YPB: Yellow pea bread
YPC: Yellow pea chips
YPP: Yellow pea pasta
