# Supplementary material for: Effects of reduced gluten intake through the inclusion of yellow pea-based products on abdominal symptoms in habitual wheat consumers: a non-randomized clinical trial
Source: Eur J Nutr. 2026 Aug 29;65(6):246. doi: 10.1007/s00394-026-04097-2 (PMC13525955; doi:10.1007/s00394-026-04097-2)
Supplement: Supplementary file 1 [file 394_2026_4097_MOESM1_ESM.docx]

Supplementary Information

**Effects of reduced gluten intake through the inclusion of yellow pea–based products on abdominal symptoms in habitual wheat consumers: A non-randomized clinical trial**

Hitoshi Shimbo¹^, a^, Mamoru Ito^1, b^, Yuto Aoki^1, c^, Joto Yoshimoto^1^, Mikiya Kishi¹^, d^, Jiro Saito^2^, Yukihiro Ohya^3, 4, e^ Masashi Nakamura^3, 4^ Senju Hashimoto^4, 5^, and Akiko Yagami ^3, 4^

^1^ Central Research Institute, Mizkan Holdings Co., Ltd., Handa-Shi, Japan

^2^ Medical Station Clinic Meguro-ku, Japan

^3^ Department of Allergology, Fujita Health University School of Medicine, Nagoya, Aichi, Japan

^4^ Fujita Health University General Allergy Center, Nagoya, Aichi, Japan.

^5^ Department of Gastroenterology, Fujita Health University Bantane Hospital, Nagoya, Aichi, Japan.

^a^ ORCID; 0009-0008-9412-1415

^b^ ORCID; 0000-0002-4780-3930

^c^ ORCID; 0009-0001-4682-6831

^d^ ORCID; 0000-0002-5995-9094

^e^ ORCID; 0000-0002-1560-3942

*** Corresponding author** (email: hitoshi_shimbo@mizkan.co.jp)

Supplementary Table1. Vital Signs and Body Composition

|  |  | Week 0 | | *p¹* |  | Week 4 | | *p*^2^ | *p³* |
| --- | --- | --- | --- | --- | --- | --- | --- | --- | --- |
| Body Weight (kg) | AS– | 60.52 | (55.32, 65.73) | 0.53 |  | 60.64 | (55.23, 66.04) | 0.62 | 0.80 |
|  | AS+ | 58.50 | (54.07, 62.93) |  |  | 58.96 | (54.11, 63.81) |  | 0.32 |
| BMI (kg/m^2^) | AS– | 21.42 | (20.27, 22.57) | 0.61 |  | 21.45 | (20.23, 22.67) | 0.77 | 0.86 |
|  | AS+ | 21.07 | (20.15, 21.99) |  |  | 21.22 | (20.09, 22.35) |  | 0.37 |
| SBP (mmHg) | AS– | 115.21 | (105.38, 125.05) | 0.22 |  | 113.86 | (106.53, 121.18) | 0.45 | 0.63 |
|  | AS+ | 108.73 | (103.49, 113.97) |  |  | 110.27 | (103.33, 117.21) |  | 0.49 |
| DBP (mmHg) | AS– | 68.00 | (62.05, 73.95) | 0.91 |  | 67.86 | (63.33, 72.38) | 0.74 | 0.94 |
|  | AS+ | 67.60 | (62.81, 72.39) |  |  | 68.93 | (63.59, 74.28) |  | 0.32 |
|  | AS+ | 67.27 | (62.51, 72.02) |  |  | 72.80 | (67.78, 77.82) |  | 0.02* |

Data are presented as means (95% confidence intervals). ^1, 2^ For normally distributed data, between-group comparisons were performed using an unpaired t-test, whereas for non-normally distributed data, the Wilcoxon rank-sum test was used. ^3^ For normally distributed data, within-group comparisons (week 0 vs. week 4) were performed using a paired t-test, whereas for non-normally distributed data, the Wilcoxon signed-rank test was used. * p < 0.05.

AS–, participants without abdominal symptoms; AS+, participants with abdominal symptoms; BMI, body mass index; SBP, systolic blood pressure; DBP, diastolic blood pressure.

Supplementary Table 2. Effect of Gluten Reduction on Clinical Symptoms

|  |  | Week 0 | | *p¹* |  | Week 2 | | *p*^2^ | *p³* |  | Week 4 | | *p⁴* | *p⁵* |
| --- | --- | --- | --- | --- | --- | --- | --- | --- | --- | --- | --- | --- | --- | --- |
| Abdominal pain or discomfort | AS- | 0.64 | (-0.09, 1.38) | <0.001*** |  | 0.50 | (0.01, 0.99) | <0.001*** | 1.000 |  | 0.43 | (-0.01, 0.87) | <0.001*** | 0.656 |
|  | AS+ | 5.13 | (3.83, 6.44) |  |  | 2.40 | (1.65, 3.15) |  | 0.003** |  | 2.73 | (1.49, 3.98) |  | 0.021* |
| Abdominal distension | AS- | 0.86 | (0.15, 1.57) | <0.001*** |  | 0.86 | (0.31, 1.41) | 0.063 | 1.000 |  | 0.86 | (0.36, 1.36) | 0.525 | 0.938 |
|  | AS+ | 4.67 | (3.30, 6.03) |  |  | 2.13 | (1.07, 3.20) |  | 0.002** |  | 1.40 | (0.52, 2.28) |  | <0.001*** |
| Hard stools | AS- | 1.36 | (0.30, 2.41) | 0.003** |  | 1.43 | (0.17, 2.68) | 0.054 | 1.000 |  | 1.86 | (0.56, 3.16) | 0.871 | 0.375 |
|  | AS+ | 4.67 | (3.06, 6.27) |  |  | 3.53 | (1.90, 5.17) |  | 0.115 |  | 1.93 | (0.82, 3.05) |  | 0.002** |
| Urgent need for defecation | AS- | 0.57 | (0.03, 1.11) | 0.007** |  | 0.79 | (0.10, 1.47) | 0.141 | 0.750 |  | 0.79 | (0.00, 1.58) | 0.845 | 0.656 |
|  | AS+ | 3.27 | (1.55, 4.99) |  |  | 2.40 | (0.86, 3.94) |  | 0.395 |  | 1.07 | (-0.01, 2.14) |  | 0.011* |
| Feeling of incomplete evacuation | AS- | 1.14 | (0.08, 2.20) | 0.007** |  | 1.36 | (0.43, 2.28) | 0.198 | 0.844 |  | 1.14 | (0.21, 2.07) | 0.141 | 0.820 |
|  | AS+ | 3.87 | (2.08, 5.65) |  |  | 2.40 | (1.13, 3.67) |  | 0.170 |  | 2.07 | (0.89, 3.24) |  | 0.043* |
| Loose stools | AS- | 1.00 | (0.12, 1.88) | 0.039* |  | 0.36 | (-0.01, 0.72) | 0.005** | 0.219 |  | 0.86 | (-0.07, 1.79) | 0.083 | 0.807 |
|  | AS+ | 3.13 | (1.61, 4.66) |  |  | 1.93 | (1.03, 2.83) |  | 0.147 |  | 1.87 | (0.84, 2.89) |  | 0.092 |
| Nausea and vomiting | AS- | 0.00 | (0.00, 0.00) | 0.100 |  | 0.07 | (-0.08, 0.23) | 0.265 | 1.000 |  | 0.14 | (-0.07, 0.35) | 0.220 | 0.500 |
|  | AS+ | 0.60 | (-0.09, 1.29) |  |  | 0.40 | (-0.01, 0.81) |  | 0.813 |  | 0.80 | (-0.09, 1.69) |  | 0.750 |
| Decreased passage of stools | AS- | 1.64 | (0.81, 2.48) | 0.198 |  | 1.50 | (0.33, 2.67) | 0.237 | 0.531 |  | 1.71 | (0.49, 2.94) | 0.800 | 1.000 |
|  | AS+ | 2.73 | (1.52, 3.94) |  |  | 1.87 | (1.15, 2.59) |  | 0.059 |  | 1.67 | (0.81, 2.52) |  | 0.060 |
| Increased passage of stools | AS- | 1.07 | (0.92, 1.23) | 0.842 |  | 1.21 | (0.65, 1.78) | 0.662 | 0.754 |  | 1.07 | (0.27, 1.87) | 0.386 | 1.000 |
|  | AS+ | 1.20 | (0.68, 1.72) |  |  | 1.07 | (0.68, 1.46) |  | 0.795 |  | 1.87 | (0.37, 3.36) |  | 0.684 |
| Dermatitis | AS- | 0.86 | (0.31, 1.41) | 0.174 |  | 0.29 | (-0.07, 0.64) | 0.409 | 0.031* |  | 0.64 | (0.06, 1.22) | 0.684 | 0.406 |
|  | AS+ | 0.60 | (-0.21, 1.41) |  |  | 0.80 | (0.01, 1.59) |  | 1.000 |  | 0.60 | (-0.09, 1.29) |  | 0.938 |
| Headache | AS- | 0.29 | (-0.07, 0.64) | 0.119 |  | 0.07 | (-0.08, 0.23) | 0.040* | 0.500 |  | 0.14 | (-0.07, 0.35) | 0.354 | 0.625 |
|  | AS+ | 1.27 | (0.21, 2.32) |  |  | 0.93 | (0.19, 1.67) |  | 0.500 |  | 0.67 | (-0.05, 1.38) |  | 0.063 |
| Foggy mind | AS- | 0.21 | (-0.12, 0.55) | 0.110 |  | 0.07 | (-0.08, 0.23) | 0.046* | 0.750 |  | 0.21 | (-0.12, 0.55) | 0.108 | 1.000 |
|  | AS+ | 1.33 | (0.14, 2.53) |  |  | 1.13 | (0.18, 2.09) |  | 0.582 |  | 1.47 | (0.21, 2.72) |  | 0.766 |
| Fatigue | AS- | 2.29 | (1.40, 3.18) | 0.220 |  | 1.79 | (0.87, 2.70) | 0.249 | 0.254 |  | 1.71 | (0.82, 2.60) | 0.106 | 0.088 |
|  | AS+ | 3.27 | (2.04, 4.50) |  |  | 2.67 | (1.35, 3.98) |  | 0.078 |  | 3.27 | (1.87, 4.66) |  | 0.707 |
| Joint muscle pains | AS- | 0.36 | (-0.07, 0.79) | 0.222 |  | 0.36 | (-0.01, 0.72) | 0.827 | 1.000 |  | 0.57 | (0.03, 1.11) | 0.645 | 0.750 |
|  | AS+ | 1.20 | (0.17, 2.23) |  |  | 0.60 | (-0.02, 1.22) |  | 0.188 |  | 1.00 | (0.11, 1.89) |  | 0.567 |

Data are presented as means (95% confidence intervals). ^1,2,4^ For normally distributed data, between-group comparisons were performed using an unpaired t-test, whereas the Wilcoxon rank-sum test was used for non-normally distributed data. ^3, 5^ For normally distributed data, within-group comparisons ( vs. week 0) were performed using a paired t-test, whereas for non-normally distributed data, the Wilcoxon signed-rank test was used. * p < 0.05, ** p < 0.01, *** p < 0.001

Supplementary Table 3. Effect of Gluten Reduction on Quality of Life in Participants Consuming Less than 10% Intake of Wheat-Based Foods

|  |  | Week 0 | | *p¹* |  | Week 4 | | *p²* | *p³* |
| --- | --- | --- | --- | --- | --- | --- | --- | --- | --- |
| PCS | AS– | 54.28 | (51.72, 56.84) | 0.778 |  | 55.76 | (53.71, 57.81) | 0.118 | 0.042* |
|  | AS+ | 53.65 | (49.57, 57.73) |  |  | 52.95 | (49.82, 56.08) |  | 0.517 |
| MCS | AS– | 58.44 | (54.15, 62.72) | 0.019* |  | 59.11 | (55.61, 62.61) | 0.087 | 0.553 |
|  | AS+ | 51.09 | (46.42, 55.75) |  |  | 53.19 | (46.98, 59.39) |  | 0.350 |
| RCS | AS– | 51.88 | (49.41, 54.36) | 0.604 |  | 50.38 | (48.57, 52.18) | 0.696 | 0.127 |
|  | AS+ | 50.59 | (45.90, 55.29) |  |  | 51.24 | (46.92, 55.56) |  | 0.702 |
| Physical functioning | AS– | 96.54 | (93.96, 99.12) | 0.896 |  | 97.31 | (95.74, 98.88) | 0.310 | 0.625 |
|  | AS+ | 95.71 | (92.34, 99.08) |  |  | 95.00 | (92.00, 98.00) |  | 0.750 |
| Role physical | AS– | 96.16 | (88.85, 103.47) | 0.156 |  | 97.12 | (92.88, 101.37) | 0.176 | 1.000 |
|  | AS+ | 91.52 | (85.26, 97.79) |  |  | 92.42 | (85.61, 99.23) |  | 0.688 |
| Bodily pain | AS– | 89.15 | (80.12, 98.18) | 0.143 |  | 91.85 | (86.08, 97.61) | 0.050* | 0.500 |
|  | AS+ | 77.29 | (65.28, 89.29) |  |  | 78.50 | (67.80, 89.20) |  | 0.724 |
| General health | AS– | 76.85 | (69.36, 84.34) | 0.026* |  | 77.38 | (70.14, 84.63) | 0.131 | 0.753 |
|  | AS+ | 63.71 | (54.32, 73.11) |  |  | 67.14 | (55.01, 79.27) |  | 0.406 |
| Vitality | AS– | 69.25 | (58.41, 80.10) | 0.182 |  | 72.13 | (62.07, 82.19) | 0.209 | 0.293 |
|  | AS+ | 59.84 | (49.67, 70.02) |  |  | 63.41 | (52.75, 74.08) |  | 0.449 |
| Social functioning | AS– | 99.04 | (96.94, 101.13) | 0.016* |  | 92.31 | (83.30, 101.32) | 0.890 | 0.125 |
|  | AS+ | 84.82 | (73.44, 96.21) |  |  | 91.96 | (84.16, 99.77) |  | 0.188 |
| Role emotional | AS– | 92.95 | (86.19, 99.72) | 0.566 |  | 94.24 | (88.65, 99.82) | 0.568 | 0.500 |
|  | AS+ | 88.10 | (78.91, 97.29) |  |  | 87.50 | (76.22, 98.78) |  | 0.844 |
| Mental health | AS– | 83.46 | (76.66, 90.26) | 0.013* |  | 84.62 | (79.61, 89.62) | 0.052 | 0.650 |
|  | AS+ | 71.43 | (64.14, 78.72) |  |  | 73.21 | (62.44, 83.99) |  | 0.597 |

Data are presented as mean (95% confidence intervals). ^1,2^ For normally distributed data, between-group comparisons were performed using an unpaired t-test, whereas for non-normally distributed data, the Wilcoxon rank-sum test was used. ^3^ For normally distributed data, within-group comparisons (week 0 vs. week 4) were performed using a paired t-test, whereas for non-normally distributed data, the Wilcoxon signed-rank test was used. * p < 0.05.

AS–, participants without abdominal symptoms; AS+, participants with abdominal symptoms; PCS, Physical Component Summary; MCS, Mental Component Summary; RCS, Role/Social Component Summary.

Supplementary Table 4. Effect of Gluten Reduction on α-Diversity

|  |  | Week 0 | | *p¹* |  | Week 4 | | *p²* | *p³* |
| --- | --- | --- | --- | --- | --- | --- | --- | --- | --- |
| Shannon index | AS– | 6.17 | (5.88, 6.47) | 0.48 |  | 5.88 | (5.64, 6.12) | 0.42 | 0.10 |
|  | AS+ | 6.13 | (5.86, 6.39) |  |  | 6.04 | (5.70, 6.37) |  | 0.68 |
| Observed OTUs | AS– | 196.94 | (168.14, 225.73) | 0.47 |  | 183.52 | (162.01, 205.03) | 0.17 | 0.40 |
|  | AS+ | 212.94 | (176.37, 249.51) |  |  | 209.13 | (176.93, 241.33) |  | 0.88 |
| Faith’s phylogenetic diversity | AS– | 6.17 | (5.88, 6.47) | 0.21 |  | 5.88 | (5.64, 6.12) | 0.18 | 0.53 |
|  | AS+ | 6.13 | (5.86, 6.39) |  |  | 6.04 | (5.70, 6.37) |  | 0.56 |

Data are presented as means (95% confidence intervals). ^1,2^ For normally distributed data, between-group comparisons were performed using an unpaired t-test, whereas for non-normally distributed data, the Wilcoxon rank-sum test was used. ^3^ For normally distributed data, within-group comparisons (week 0 vs. week 4) were performed using a paired t-test, whereas for non-normally distributed data, the Wilcoxon signed-rank test was used.

AS–, participants without abdominal symptoms; AS+, participants with abdominal symptoms; OTUs, operational taxonomic units.

Supplementary Table 5. Effect of Gluten Reduction on Relative Abundance of Gut Microbiota

|  |  | Week 0 | | *p¹* |  | Week 4 | | *p²* | *p³* |  | Change | | *p⁴* |
| --- | --- | --- | --- | --- | --- | --- | --- | --- | --- | --- | --- | --- | --- |
| *Oscillospiraceae* | AS– | 0.203 | (0.089, 0.316) | 0.039* |  | 0.072 | (0.031, 0.114) | 0.247 | 0.030* |  | –0.130 | (–0.228, –0.033) | 0.019* |
|  | AS+ | 0.064 | (–0.005, 0.133) |  |  | 0.048 | (0.003, 0.093) |  | 1.000 |  | –0.016 | (–0.083, 0.051) |  |
| *Lachnospiraceae_NK4A136_group; unclassified* | AS– | 0.016 | (0.000, 0.033) | 0.007** |  | 0.272 | (0.018, 0.527) | 0.615 | 0.014* |  | 0.256 | (0.001, 0.511) | 0.390 |
|  | AS+ | 0.241 | (0.039, 0.442) |  |  | 0.335 | (0.094, 0.576) |  | 0.401 |  | 0.095 | (–0.140, 0.329) |  |
| *Negativibacillus; unclassified* | AS– | 0.001 | (–0.001, 0.003) | 0.464 |  | 0.013 | (0.001, 0.025) | 0.178 | 0.047* |  | 0.012 | (0.000, 0.024) | 0.047* |
|  | AS+ | 0.011 | (–0.004, 0.027) |  |  | 0.010 | (–0.005, 0.025) |  | 1.000 |  | –0.001 | (–0.003, 0.001) |  |
| *Eggerthellaceae; unclassified* | AS– | 0.011 | (–0.011, 0.034) | 0.127 |  | 0.013 | (–0.010, 0.036) | 0.601 | 0.625 |  | 0.002 | (–0.001, 0.004) | 0.016* |
|  | AS+ | 0.040 | (0.000, 0.081) |  |  | 0.010 | (0.000, 0.020) |  | 0.301 |  | –0.030 | (–0.062, 0.002) |  |
| *Muribaculaceae; unclassified* | AS– | 0.861 | (–0.167, 1.890) | 0.042* |  | 1.141 | (–0.084, 2.367) | 0.042* | 0.125 |  | 0.280 | (–0.056, 0.616) | 0.042* |
|  | AS+ | 0.000 | (0.000, 0.000) |  |  | 0.000 | (0.000, 0.000) |  | NA |  | 0.000 | (0.000, 0.000) |  |
| *Lachnospiraceae; unclassified* | AS– | 0.017 | (0.002, 0.032) | 0.277 |  | 0.002 | (–0.003, 0.008) | 0.531 | 0.063 |  | –0.014 | (–0.029, 0.000) | 0.017* |
|  | AS+ | 0.010 | (–0.006, 0.025) |  |  | 0.019 | (–0.015, 0.054) |  | 1.000 |  | 0.009 | (–0.011, 0.030) |  |
| *Ruminococcaceae; unclassified* | AS– | 0.046 | (–0.006, 0.098) | 0.052 |  | 0.036 | (–0.006, 0.079) | 0.398 | 0.938 |  | –0.010 | (–0.029, 0.010) | 0.049* |
|  | AS+ | 0.078 | (0.025, 0.132) |  |  | 0.022 | (0.005, 0.039) |  | 0.050 |  | –0.056 | (–0.113, 0.001) |  |
| *Lachnospiraceae; unclassified; human.gut.metagenome* | AS– | 0.037 | (–0.009, 0.083) | 0.100 |  | 0.022 | (–0.011, 0.055) | 0.930 | 0.625 |  | –0.015 | (–0.033, 0.003) | 0.035* |
|  | AS+ | 0.000 | (0.000, 0.000) |  |  | 0.025 | (–0.009, 0.059) |  | 0.250 |  | 0.025 | (–0.009, 0.059) |  |
| *Butyricimonas; unclassified* | AS– | 0.053 | (0.010, 0.096) | 0.942 |  | 0.026 | (0.001, 0.052) | 0.101 | 0.313 |  | –0.027 | (–0.062, 0.008) | 0.015* |
|  | AS+ | 0.065 | (0.007, 0.123) |  |  | 0.118 | (0.035, 0.202) |  | 0.287 |  | 0.053 | (0.004, 0.102) |  |
| *Holdemanella* | AS– | 0.771 | (–0.455, 1.997) | 0.215 |  | 0.622 | (–0.405, 1.649) | 0.732 | 0.813 |  | –0.149 | (–0.365, 0.067) | 0.032* |
|  | AS+ | 0.172 | (–0.197, 0.541) |  |  | 0.174 | (–0.199, 0.547) |  | 0.750 |  | 0.003 | (–0.002, 0.007) |  |
| *Defluviitaleaceae UCG011; unclassified* | AS– | 0.000 | (0.000, 0.000) | 0.017* |  | 0.000 | (0.000, 0.000) | 0.483 | NA |  | 0.000 | (0.000, 0.000) | 0.017* |
|  | AS+ | 0.013 | (0.000, 0.025) |  |  | 0.004 | (–0.002, 0.011) |  | 0.250 |  | –0.009 | (–0.018, 0.001) |  |
| *Eisenbergiella; unclassified* | AS– | 0.015 | (–0.003, 0.032) | 0.182 |  | 0.079 | (0.000, 0.159) | 0.531 | 0.064 |  | 0.065 | (–0.015, 0.144) | 0.030* |
|  | AS+ | 0.096 | (–0.018, 0.210) |  |  | 0.113 | (–0.026, 0.251) |  | 0.695 |  | 0.017 | (–0.156, 0.189) |  |
| *Eubacterium hallii group* | AS– | 0.065 | (0.000, 0.130) | 0.336 |  | 0.062 | (–0.001, 0.126) | 0.809 | 1.000 |  | –0.003 | (–0.010, 0.004) | 0.047* |
|  | AS+ | 0.030 | (–0.016, 0.076) |  |  | 0.050 | (–0.003, 0.103) |  | 0.688 |  | 0.020 | (–0.005, 0.044) |  |
| *Eubacterium hallii group; unclassified* | AS– | 0.608 | (0.327, 0.888) | 0.330 |  | 0.567 | (0.232, 0.902) | 0.380 | 0.862 |  | –0.040 | (–0.356, 0.275) | 0.019* |
|  | AS+ | 0.481 | (0.158, 0.804) |  |  | 0.865 | (0.435, 1.296) |  | 0.179 |  | 0.384 | (0.089, 0.680) |  |
| *Oscillospiraceae UCG005* | AS– | 0.000 | (0.000, 0.000) | 0.042* |  | 0.005 | (–0.002, 0.013) | 0.933 | 0.500 |  | 0.005 | (–0.002, 0.013) | 0.015* |
|  | AS+ | 0.042 | (0.002, 0.082) |  |  | 0.020 | (–0.011, 0.051) |  | 0.419 |  | –0.022 | (–0.041, –0.002) |  |
| *Incertae Sedis* | AS– | 0.013 | (–0.007, 0.033) | 0.004** |  | 0.053 | (–0.049, 0.155) | 0.010* | 0.875 |  | 0.040 | (–0.046, 0.125) | 0.023* |
|  | AS+ | 0.186 | (0.029, 0.343) |  |  | 0.134 | (–0.009, 0.278) |  | 0.244 |  | –0.052 | (–0.116, 0.013) |  |
| *Phocea; unclassified* | AS– | 0.014 | (0.001, 0.028) | 0.027* |  | 0.001 | (–0.001, 0.003) | 0.741 | 0.094 |  | –0.013 | (–0.026, –0.001) | 0.019* |
|  | AS+ | 0.001 | (–0.001, 0.002) |  |  | 0.001 | (–0.001, 0.002) |  | 1.000 |  | 0.000 | (–0.002, 0.002) |  |
| *Megasphaera* | AS– | 0.230 | (–0.033, 0.494) | 0.175 |  | 0.145 | (–0.009, 0.299) | 0.233 | 0.813 |  | –0.085 | (–0.210, 0.040) | 0.025* |
|  | AS+ | 0.069 | (–0.079, 0.217) |  |  | 0.092 | (–0.105, 0.289) |  | 1.000 |  | 0.023 | (–0.026, 0.072) |  |
| *Bacteroides dorei* | AS– | 3.254 | (0.578, 5.930) | 0.750 |  | 6.583 | (1.633, 11.532) | 0.887 | 0.413 |  | 3.329 | (0.951, 5.708) | 0.021* |
|  | AS+ | 5.724 | (0.910, 10.539) |  |  | 5.070 | (1.099, 9.042) |  | 0.855 |  | –0.654 | (–2.707, 1.399) |  |

Data are presented as means (95% confidence intervals). ^1,2,4^ For normally distributed data, between-group comparisons were performed using an unpaired t-test, whereas the Wilcoxon rank-sum test was used for non-normally distributed data. ^3^ For normally distributed data, within-group comparisons (week 0 vs. week 4) were performed using a paired t-test, whereas for non-normally distributed data, the Wilcoxon signed-rank test was used. * p < 0.05, ** p < 0.01, *** p < 0.001.
